# Leadership in the context of digital health services: A concept analysis

**DOI:** 10.1111/jonm.13763

**Published:** 2022-08-25

**Authors:** Elina Laukka, Tarja Pölkki, Outi Kanste

**Affiliations:** ^1^ Research Unit of Nursing Science and Health Management University of Oulu Oulu Finland; ^2^ Medical Research Center Oulu University Hospital and University of Oulu Oulu Finland

**Keywords:** concept analysis, digital, health care, leadership, telemedicine

## Abstract

**Aim:**

To define and clarify the concept of leadership in the context of digital health services using Walker's and Avant's concept analysis model.

**Background:**

Conceptualizing leadership in the context of digital health services is needed to deliver higher quality services and advance research.

**Method:**

Searches were conducted of MEDLINE (Ovid), Scopus, CINAHL (EBSCO) and ProQuest (ABI/INFORM). Empirical articles were included if they reported *attributes*, *antecedents* or *consequences* of leadership in the study context. A total of 4037 references were identified; 23 were included.

**Results:**

Leadership attributes concerned leaders' behaviour, roles and qualities. Antecedents concerned informatics skills and competence, information and tools, understanding care systems and their complexity and education. Consequences related to organization, professionals and patient and care.

**Conclusion:**

Based on our results, the term ‘e‐leadership’ should be more widely utilized in nursing practice and research.

**Implications for nursing management:**

Nurse leaders need to be strong leaders; they need to be visionary and use strategic thinking to develop existing and new digital solutions. By becoming e‐leaders, nurse leaders may increase the successful development and implementation of eHealth and benefit clinicians and patients.

## 1 INTRODUCTION

Digital health care is important because its meaningfulness has often been emphasized due to problems in modern health care, such as increasing costs (De La Torre‐Diéz et al., 2015) and COVID‐19 (Wind et al., 2020). For example, hospitals with electronic health records (EHRs) with basic capabilities (EHRs) have a 12% lower average cost than those hospitals that do not have EHR (Highfill, 2019). However, despite their potential and heavy investment, implementation of digital health services often fails (Herrmann et al., 2018; Öberg et al., 2018), with poor leadership cited as one reason (Abbott et al., 2014; Mair et al., 2012); leaders often seem ill‐prepared to handle future challenges (Day, 2000), and it may be that leaders are not conscious of what leadership actually is in the context of digital health services.

Although health care leaders traditionally lead clinical health services (Sood et al., 2017), leadership develops over time and in different contexts (Day, 2000). Recently, health care leadership has been transformed by digital services; nurse leaders' responsibilities have expanded into digitalizing health care (Cowan, 2014; Sandström et al., 2011) and even artificial intelligence (Chen & Decary, 2020). As Cowan (2014) noted, nurse leaders have more commonly been tasked with coordinating digital health care, and their role seems to be more emphasized in leading digital health services than physician leaders (Keijser et al., 2016). According to Strudwick, Nagle, Morgan, et al. (2019), leaders were likely unaware of the gaps they have in their informatics knowledge and skills. In addition, the literature provides little clarity as to what leadership in the context of digital health services entails (Lulu, 2019; Tremblay, 2017), and it has been suggested that research should focus on continuously transforming leadership (Dickson, 2009). Because of the lack of clarity about leadership in digital health services, Walker and Avant's (2019) method of concept analysis was used to identify the attributes, antecedents, consequences and empirical referents of this concept. Understanding the concept of leadership in the context of digital health services would be important, since it may ease leaders to obtain required competencies and behaviours related to information and communication technology (ICT) acquisition and use (Strudwick, Nagle, Morgan, et al., 2019).

## 2 BACKGROUND

Health care organizations have been recognized as complex (Begun & Thygeson, 2015): they have been scrutinized as complex adaptive systems (CASs) that can self‐organize, adapt and learn (Paina & Peters, 2012). Digitalization transforms the context in which leaders operate (Hernez‐Broome & Hughes, 2004). High levels of digitalization tend to make health care organizations even more challenging to lead: every new digital service requires decision‐making, implementation, assessment and secure usage among end‐users. Avolio et al. (2014) have suggested that organizational structures, including leadership, may transform due to the implementation of Advanced Information Technology (AIT). Thus, every digital health solution may be understood as a complex innovation, which according to Chuang et al. (2012) means that *the implementation process requires systematic organizational changes in structure, staffing, workflows, and/or policies, as well as coordinated innovation use by multiple organizational members*—all of which concern leaders. Digital innovations then again may transform the work of professionals and enable virtual or geographically dispersed units and teams (Hernez‐Broome & Hughes, 2004). The interdisciplinary literature shows that leadership in digital environment requires different kinds of leadership behaviours such as transformational behaviour, strategic‐oriented behaviour and servant leadership (Avolio et al., 2014; Larjovuori et al., 2016).

Successful implementation of digital services would be important since their costs are extremely high. For example, in the United Kingdom EHR implementation costs £200 million (EUR 275 million, USD 293 million), and in Denmark DKK 2.8 billion (EUR 375 million, USD 400 million). The implementation of the new EHR has not been trouble‐free in the United Kingdom or Denmark, and in Norway, managers have been recognized to play an important part in the implementation process (Hertzum & Ellingsen, 2019). According to a scoping review by Laukka et al. (2020), health care leaders need to adopt certain behaviours to support the implementation of health information technology; leaders need to act as supporters, change managers, advocates, project managers, decision‐makers, facilitators and champions. Another review shows that leaders also need several informatics competencies such as informatics knowledge, informatics skills and computer skills (Strudwick, Nagle, Kassam, et al., 2019).

However, leaders cannot fully act in expected roles since their understanding of digitalization and its implementation may not be any better than their subordinates' understanding of it (Laukka et al., 2020). This may be because there is a lack of understanding about leadership in the context of digital health services, and thus, health care leaders may be ill‐prepared to lead in a transformed digital environment. As it appears, prior reviews synthesized leadership roles and competencies but making the most of HIT requires proper health care leadership in other processes as well (Simpson, 2004). For a health care leader managing technology is an issue about the three ‘Ps’: People, processes and (computer) programmes (Simpson, 2004).

Previous concept analyses have scrutinized health care leadership, for example, in terms of implementation leadership (Castiglione, 2020), transformational leadership (Fischer, 2016) and succession planning (Titzer & Shirey, 2013). Since research on leadership in this context is limited and developing transforming leadership is currently challenged by rapid digital innovation (Dickson, 2009), conceptualizing leadership may provide guidance for service development and future research. The conceptualization of leadership in the context of digital health services provides a better understanding of today's health care leadership and how it supports digitalization and improves implementation. In addition, conceptualization may facilitate further research and thus help reshape existing and emerging leadership models. Precise conceptualization of leadership in the context of digital health services is therefore needed to support leaders working on the frontline and at middle and senior management levels, to improve digital health services, facilitate evolving leadership and advance research. Thus, the aim of this paper was to define and clarify the concept of leadership in the context of digital health services using Walker's and Avant's concept analysis model.

## 3 METHODS

The detailed protocol for this concept analysis has been published (Laukka et al., 2021). We used the concept analysis model by Walker and Avant (2019), a frequently used tool in health care settings (Nuopponen, 2010). To identify all relevant literature about leadership in the context of digital health services, a literature review was conducted in accordance with the Joanna Briggs Institute's (JBI) search protocol for scoping reviews (Peters, Godfrey, et al., 2020).

### 3.1 Eligibility criteria

A participants, concept and context (PCC) framework was applied when defining eligibility criteria (Table 1).

**TABLE 1:** Eligibility criteria

Eligibility	Participants	Concept	Context	Language	Article type
Inclusion	Studies on health care line managers regardless of their position or health care field	Health care/service leadership	Digital health services	English, Finnish, Swedish	Empirical studies
Exclusion	Studies on leaders working solely with IT management	Not related to health care/service leadership	Health services with no digitalization of any kind pharmacy radiology		Reviews, anecdotal papers without references

Peer‐reviewed empirical studies were considered. The study participants comprised all positional health care leaders; we included nursing and physician leaders, since one may safely generalize about leadership in the context of digital health services (Keijser et al., 2016). Publications eligible for inclusion must either define or clarify the concept of leadership in the relevant context.

### 3.2 Search strategy

A three‐step search strategy was used to retrieve both published and unpublished studies. An information specialist helped develop the initial and final search strategies. An initial limited search of MEDLINE (Ovid) was undertaken on 19 October 2020 as part of a concept analysis protocol (Laukka et al., 2021). On 31 November 2020, four databases (MEDLINE (Ovid), Scopus, CINAHL (EBSCO) and ProQuest (ABI/INFORM)) were searched using indexing and keywords with a limit of 10 years (Appendix S1). Keywords were truncated where appropriate.

### 3.3 Screening and synthesis

**FIGURE 1:**
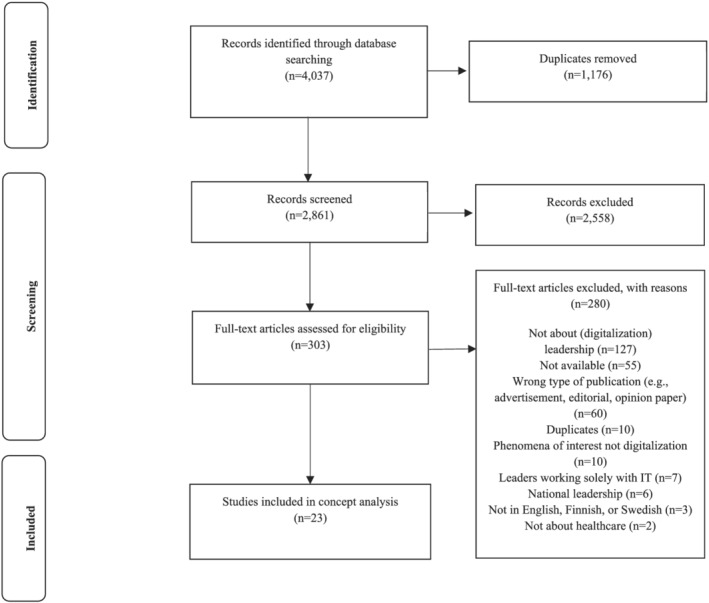
PRISMA flow diagram adapted for scoping review

The Covidence systematic review software package (v2422) was used for screening purposes and removing duplicates. Two team members (EL & OK) screened the articles independently following the eligibility criteria. After title and abstract screening, potentially relevant studies were retrieved in full. Disagreements were resolved through consensus. Search and screening results are presented in a PRISMA flow diagram (Figure 1) (Peters, Marnie, et al., 2020).

Searching returned 4037 possible records. After removing 1176 duplicates, 2861 records underwent title and abstract screening, which included 2558 papers that did not meet the inclusion criteria. Full‐text assessment for eligibility was performed on 303 papers, of which 23 met the inclusion criteria (Figure 1).

Extraction was undertaken by one researcher (EL) and confirmed by another (OK). Data included publishing information, design, purpose, sample and instrument (if mentioned). Extraction also included main findings and stated definitions of leadership in the context of digital health services, attributes, antecedents and consequences (Walker & Avant, 2019) (Table 2 and Appendix S2). Similar themes and trends were discovered and grouped iteratively by the end of data collection (Appendix S2).

**TABLE 2:** Summary of the research studies

Reference (country of origin for scientific studies and case studies)	Design	Purpose	Sample	Instrument	Findings	Term used	Context	Selected definitions
Ahonen et al. (2016) (Finland)	Mixed method	To describe nurses' contribution to the national strategy concerning eHealth development and implementation in health and social care.	10 experts representing eHealth and health informatics	Expert group questionnaire	Knowledge‐based management and active utilization of data warehouses are needed to ensure quality and safe care	Nursing leaders	eHealth services	N/A
Ali et al. (2017) (Malaysia/New Zealand)	Quantitative study	To develop a knowledge management systems success model for health care organizations.	263 doctors	Cross‐sectional survey	Leadership is a key element in promoting the success of knowledge management	Leadership	Information technology	N/A
Amlung et al. (2020) (USA)	Qualitative study	To identify recurrent themes, insights and process recommendations from stakeholders in US organizations during the health information technology (HIT) modernization of an existing electronic health record (EHR) to a commercial‐off‐the‐shelf product in both resource‐plentiful settings and in a resource‐constrained environment, the US Indian Health Service.	13 leaders	Semi‐structured interviews	Clinical and leadership involvement are important in HIT implementation	Leadership	Health information technology	N/A
Andreassen et al. (2015) (Norway)	Case study	To investigate whether there are benefits of ICT innovation seen from a managerial point of view, and, if so, whether these can explain the persistence of ICT innovation projects in the sector.	21 participants of TDW project	Interviews	Management tasks were delegated to the ICT project, which thus contributed to four processes of organizational control: Allocating resources, generating and managing enthusiasm, system correction and aligning local practice and national policies	Health care management	ICT innovation	N/A
Avdagovska et al. (2020) (Canada)	Historical study	To examine the institutional decision‐making processes that shaped the development and implementation of MyChart.	423 primary and secondary sources 10 key decision makers	Interviews	Supportive leadership, project management, focused scope, appropriate technology and vendor selection and quick decision making	Leaders	Patient portal	N/A
Chen (2018) (Taiwan)	Qualitative study	To investigate how and why electronic health (eHealth) has been applied in Taiwan and to suggest implications that may inspire other countries facing similar challenges.	38 stakeholders in health care ecosystem	In depth and focus group interviews	Leader's vision, authority and management skills might influence success in health care innovation	Leader	eHealth	N/A
Collins et al. (2017) (USA)	Delphi study	To identify nursing informatics competencies perceived as relevant and requires by nurse leaders.	34 nurse leaders in round 1, 25 nurse leaders in round 2 and 41 nurse leaders in round 3	Three‐round survey	Most nurse leaders acquired HIT knowledge through on‐the‐job training	Nurse leader	Informatics competency	N/A
Collins et al. (2015) (USA)	Cross‐sectional study	To understand existing CI governance structured and provide a model with recommended roles, partnerships and councils based on perspective of nursing informatics leaders.	12 nursing informatics leaders	Interviews	Leaders were valuing, investing in and supporting interprofessional informatics	Nursing informatics leader	Clinical informatics governance	N/A
Ennis‐Cole et al. (2018) (USA)	Qualitative case study	To explore the experiences of ten physicians following a 6‐month in‐house leadership development programme.	10 physician leaders	Interviews	Physician leadership programmes distribute knowledge and create opportunities to incorporate technology	Physician leaders	IT competency	N/A
Gjellebæk et al. (2020) (Norway and Sweden)	Qualitative study	To explore middle managers' strategies that can facilitate workplace learning when introducing eHealth and new ways of providing health care services in line with the strategies of the organization.	31 participants (middle managers, employees and persons from small business)	Three focus groups with 4‐month interval	A necessity for a shift towards learning‐oriented leadership and adaptive management that emphasizes employee involvement and opportunities for learning	Middle managers	Digitalization of health care services	N/A
Hansen and Nørup (2017) (Denmark)	Quantitative study	To analyse the associations between leadership, the implementation of information and communication technology (ICT) innovations and performance. To test the extent to which general theories of leadership style and strategy hold in the context of implementing ICT innovations.	Nearly 4000 employees and local managers	Survey	Differences in leadership during the ICT implementation process have an important impact on performance after the implementation	Leadership	Electronic patient record implementation	N/A
Kairy et al. (2014) (Canada)	In‐depth case study	To examine how telerehabilitation becomes part of existing and new clinical routines and identifies factors that enable or constrain its routine use.	92 health professional, 9 managers, 22 patients and 12 family members	Focus groups and phone interviews	Managers were one of the actors either facilitating or preventing the integration of telerehabilitation in routine practices	Leadership	Telemedicine in rehabilitation	N/A
Kahn et al. (2019) (USA)	Ethnographic study	To identify the organizational factors associated with ICU telemedicine effectiveness.	ICU nurses, nurse practitioners, physicians, telemedicine facility managers and directors	Direct observation of clinical care Semi‐structured interviews (*n* = 222) Focus groups (*n* = 18) Collection of artefacts	Three domains influence ICU telemedicine effectiveness, and one is leadership (i.e., the decisions related the role of telemedicine, conflict resolution and relationship building)	Leadership	Telemedicine in ICU	The ways in which the leadership team of the telemedicine facility, ICU, and hospital system set programme goals and impact the development and implementation goals, particularly around policies, protocols, budget and conflict management.
Kolltveit et al. (2017) (Norway)	Qualitative study	To identify perceptions of health care professionals in different working settings with respect to facilitators to engagement and participation in the application of telemedicine.	24 registered nurse and 5 clinical leaders	Focus group interviews	One of the identified conditions for success in using telemedicine was committed and responsible leadership	Leader	Telemedicine in diabetes foot care	N/A
Kujala, Heponiemi, and Hilama, (2019) (Finland)	Quantitative study	To evaluate clinical leaders' eHealth competencies and training needs in two public health care organizations in Finland.	98 clinical leaders	Survey	Managing change and planning implementation are challenging to clinical leaders	Clinical leaders	eHealth competencies	N/A
Kujala, Hörhammer, et al. (2019) (Finland)	Quantitative study	To examine whether frontline leaders' positive expectations of a patient portal and perceptions of its implementation were associated with their support for the portal.	2067 health professionals and 401 frontline leaders	Online survey	The frontline leaders' perception of vision clarity had the strongest association with their own support for the portal (*ß* = .40, *P* < .001). There is also an association between leaders' view of organizational readiness and their support (*ß* = .15, *P* = .04)	Frontline leaders	Patient portal	N/A
Liebe et al. (2016) (Germany)	Quantitative study	To examine the extent to which joint intrapreneurship of clinical leaders and IT leaders as well as a distinct innovation culture mediate the effect of user participation on hospitals' IT innovativeness.	168 clinical leaders and IT leaders	Survey	The chief information officers reported a higher implementation status than the directors of nursing, which pointed to a trend for a unidirectional gradient	Clinical leaders	IT innovations	N/A
Mills et al. (2010) (USA)	Qualitative study	To examine EMR purchases in the rural landscape by examining CAHs in Iowa that are early adopters of EMRs.	15 critical access hospital leaders	Interviews	Critical access hospital leaders also viewed EMR implementation as a necessary business strategy to remain viable and improve financial performance	Leaders	Electronic medical records	N/A
Sharpp et al. (2019) (USA)	Qualitative study	To sought nurse managers' perspectives on challenges and opportunities with technology and how it may influence communication and leadership.	16 nurse managers	Interviews	Four themes emerged regarding the nurse managers' perspectives of e‐leadership and their use of information and communication technologies: (a) cannot live without it, (b) too much, too many, (c) poor onboarding education and (d) difficulty maintaining virtual relationships	e‐leadership	Information communication technology	‘e‐Leadership is a technological paradigm derived from servant leadership and trans‐formational leadership (Arnold & Sangrá, 2018; Northouse, 2010)’.
Simon et al. (2013) (USA)	Qualitative study	To characterize the experiences of hospitals that have successfully implemented CPOE.	24 participants (physicians, nurses and pharmacists)	Interviews	Successful implementation hinged on the ability of clinical leaders to address and manage perceptions and the fear of change			
Simpson (2013) (USA)	Qualitative study	To identify and validate the gaps existing between selected CNEs' self‐ascribed lived experience information technology competencies and those laid out by AONE	Seven chief nursing officers	Ethnographic interviews	Chief nursing executives found themselves limited in their ability to advocate effectively for technology needed to support nursing practice during the evaluation and selection of clinical information systems	Chief nurse executive	Informatics competencies	N/A
Strudwick, Nagle, Kassam, et al. (2019) (Canada)	Qualitative study	To investigate the role of nurse managers in supporting point‐of‐care nurses' health information technology (IT) use and identify strategies employed by nurse managers to improve adoption, while also gathering point‐of‐care nurses' perceptions of these strategies.	10 nurse managers and 12 point‐of‐care nurses	Interviews	Nurse managers adopt the role of advocate, educator and connector, using the following strategies: Communicating system updates, demonstrating use of health IT, linking staff to resources, facilitating education and providing IT oversight	Nurse managers	Adoption of health information technology	N/A
Strudwick, Booth, Bjarnadottir, et al. (2019) (Canada)	Delphi study	To obtain consensus on the informatics competencies of priority to senior Canadian nurse leaders.	53 nurse leaders	A total of 25, 24 and 23 participants completed the survey in rounds 1, 2 and 3, respectively	A core set of informatics competencies were identified	Nurse leaders	Informatics competency	N/A

## 4 RESULTS

### 4.1 Uses of the concept of leadership in the context of digital health services

According to Cambridge Dictionary (n.d., May 23), digitalization is defined as ‘to change something such as a document to a digital form (= a form that can be stored and read by computers)’. Leadership then again is defined as ‘the set of characteristics that make a good leader’, ‘the position or fact of being the leader’ and ‘the person or people in charge of an organization’. Closely related or surrogate terms for leadership were also used. Few authors provide an official definition for leadership, or its closely related terms, in the context of digital health services (Table 3).

**TABLE 3:** Closely related terms and their definitions

Closely related term	The used definition
e‐leadership	*‘E‐leadership is defined as a social influence process mediated by AIT to produce a change in attitudes, feelings, thinking, behavior, and/or performance with individuals, groups, and/or organizations’*. (Avolio et al., 2000, p 617).
Virtual leadership	*‘When an individual manages a group they do not see in person, lead a team that is dispersed geographically, or work within a team that is partially remote, they are part of the virtual workplace’*. (Dinnocenzo, 2006, p. 14)

### 4.2 Attributes, antecedents, consequences and empirical referents

**FIGURE 2:**
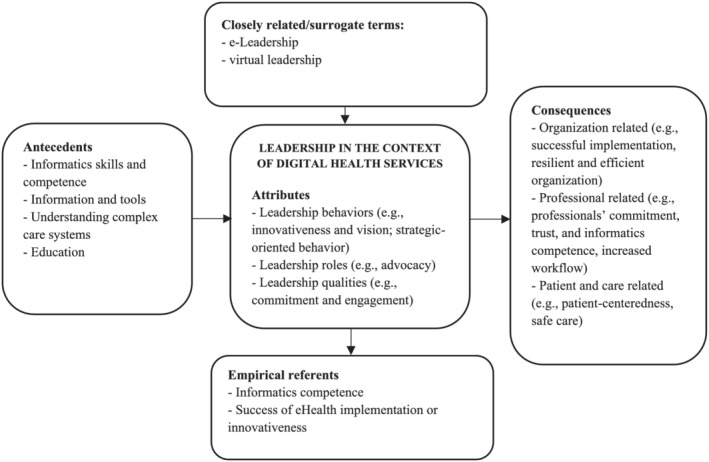
Framework of leadership in the context of digital health services

According to Walker and Avant (2019), the defining attributes of a concept are the heart of a concept analysis. We defined the key attributes of leadership in the study context and generated them as clusters. We also identified antecedents and consequences—events that occur before or because of leadership in the context of digital health services (Figure 2).

#### 4.2.1 Attributes

**FIGURE 3:**
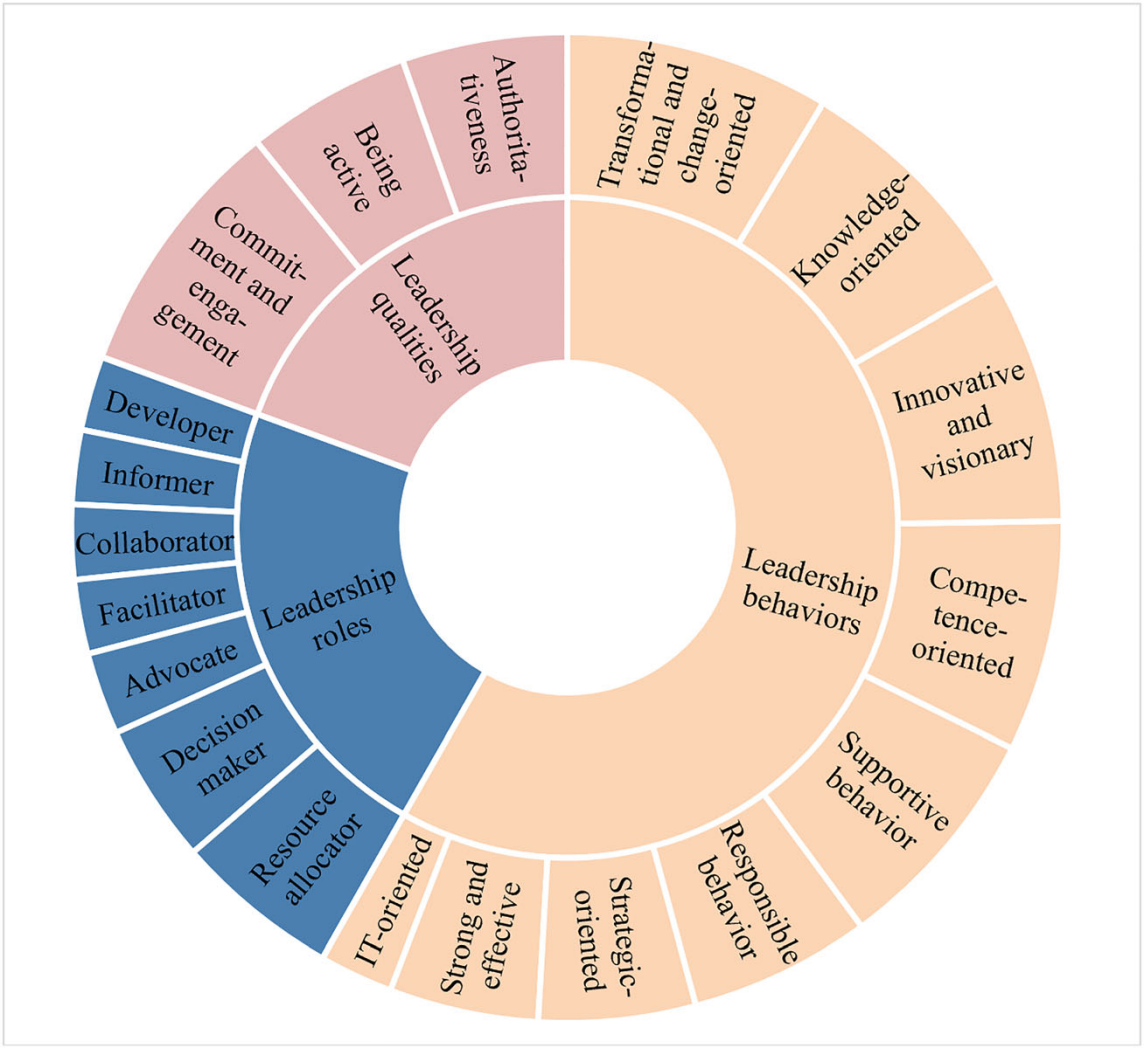
Attributes of leadership in the context of digital health services

The defining attributes associated with leadership in the study context were identified and categorized as behaviours, roles and qualities (Figure 3).

##### Leadership as a set of behaviours

The following nine attributes concerned leadership behaviour: acting as a strong and effective leader; visionary and innovative behaviour; supportive behaviour; strategic‐oriented behaviour; IT‐oriented behaviour; transformational and change‐oriented behaviour; knowledge‐oriented behaviour; responsible behaviour; and competence‐orientated behaviour.

According to earlier studies (Ahonen et al., 2016; Avdagovska et al., 2020; Gjellebæk et al., 2020; Kahn et al., 2019; Kujala, Heponiemi, & Hilama, 2019; Simon et al., 2013), leaders in the study context had to act as strong, effective leaders, meaning, for example, that they had to stand behind the implementation of digital health services. Visionary and innovative behaviour (Avdagovska et al., 2020; Chen, 2018; Ennis‐Cole et al., 2018; Kujala, Heponiemi, & Hilama, 2019; Kujala, Hörhammer, et al., 2019; Simon et al., 2013) was also seen as relevant in the implementation and development of digital health services among earlier studies. According to Kujala, Heponiemi, and Hilama (2019) and Ahonen et al. (2016), leader's clear vision associated with support for digital health services and was also seen as relevant when developing client‐centred eHealth. Several papers also highlighted the importance of supportive behaviour (Amlung et al., 2020; Avdagovska et al., 2020; Chen, 2018; Collins et al., 2017; Kahn et al., 2019; Kolltveit et al., 2017; Kujala, Hörhammer, et al., 2019; Simon et al., 2013; Strudwick, Nagle, Morgan, et al., 2019), meaning that leaders had to either provide support or create a supportive culture when professionals utilized digital services (Kujala, Hörhammer, et al., 2019). Leaders' transformational and change‐oriented behaviour broadened their leadership practices (Ali et al., 2017; Avdagovska et al., 2020; Gjellebæk et al., 2020; Hansen & Nørup, 2017; Liebe et al., 2016). Based on an included study knowledge‐oriented behaviour enabled effective interpretation of data held by organizations (Ahonen et al., 2016; Ali et al., 2017). By strategic‐orientated behaviour the included papers suggested that nurse leaders contribute to their organizations' strategic development and ensure that eHealth policies are followed (Andreassen et al., 2015; Avdagovska et al., 2020; Chen, 2018; Collins et al., 2017; Kahn et al., 2019; Kolltveit et al., 2017; Kujala, Hörhammer, et al., 2019; Simon et al., 2013; Strudwick, Nagle, Morgan, et al., 2019). Leaders' IT‐oriented behaviour included e‐leadership (Ennis‐Cole et al., 2018) and remote leadership practices (Sharpp et al., 2019). According to the included studies, competence‐orientated leaders for example recognized those who needed more education concerning eHealth (Ahonen et al., 2016; Collins et al., 2017; Sharpp et al., 2019; Simon et al., 2013; Strudwick, Nagle, Morgan, et al., 2019). Finally, leaders had to adopt responsible behaviour styles (Kolltveit et al., 2017).

##### Leadership as a set of roles

Seven attributes encapsulated leadership roles: resource allocator; decision‐maker; developer; informer; advocate; collaborator; and facilitator. Ahonen et al. (2016) suggested that leaders are responsible for allocating resources when implementing and using digital services. According to Strudwick, Booth, Bjarnadottir, et al. (2019) leaders also act as decision‐makers and select technologies to be implemented. The included paper also suggested that as informers and communicators, leaders share information in an organization (Kujala, Heponiemi, & Hilama, 2019) and also act as advocates for personnel, patients and digital health (Strudwick, Nagle, Morgan, et al., 2019). For example, leaders must stand behind the implemented digital health system, but they also need to advocate for personnel and patients when implementing or developing digital health solutions (Strudwick, Booth, Bjarnadottir, et al., 2019).

##### Leadership as a set of qualities

Three particular leadership qualities were emphasized in earlier studies: commitment and engagement (Ahonen et al., 2016; Amlung et al., 2020; Andreassen et al., 2015; Mills et al., 2010; Simpson, 2013); being active (Kolltveit et al., 2017) and authoritativeness (Chen, 2018). According to Kolltveit et al. (2017) leaders' commitment and engagement seem to be especially important: for example, there seems to be a definite need for a leader to support unequivocally the conditions that support digitalization. Among other things, leader's authoritativeness is relevant to a eHealth project's success (Chen, 2018).

#### 4.2.2 Antecedents

Four antecedents must exist before leadership in digital services can occur (Figure 2): digital health services must already exist or be on the point of introduction if leaders are to lead in this respect. According to the included studies, when leaders make decisions concerning digital health services, they need informatics skills and competence, which may for example through education (Chen, 2018; Ennis‐Cole et al., 2018; Gjellebæk et al., 2020; Kujala, Hörhammer, et al., 2019; Sharpp et al., 2019; Simpson, 2013; Strudwick, Nagle, Morgan, et al., 2019). Prior to leading digital health services, have sufficient information and tools (Kujala, Hörhammer, et al., 2019) and understand complex health care systems (Sharpp et al., 2019). Finally, according to Gjellebæk et al. (2020) and Sharpp et al. (2019) leaders need to have sufficient education to lead digital health services.

#### 4.2.3 Consequences

Leadership in the context of digital health services exhibited characteristics desirable for health care organizations, professionals and patients and their care (Figure 2). Earlier studies show that from an organizational perspective, good leadership seemed to increase the successful implementation and innovativeness of digital solutions (Ennis‐Cole et al., 2018; Gjellebæk et al., 2020; Kairy et al., 2014; Kolltveit et al., 2017; Sharpp et al., 2019), improve efficiency (Kahn et al., 2019; Mills et al., 2010) and enhance innovativeness (Chen, 2018; Liebe et al., 2016; Mills et al., 2010). When leadership in the context of digital health services is successful, it also makes organizations more resilient, allowing them to achieve goals and gain the full benefits of technology (Kujala, Hörhammer, et al., 2019; Mills et al., 2010). Due to the leadership in our context, organizations may also achieve better financial performance (Mills et al., 2010) and greater level of sustainability (Strudwick, Booth, Bjarnadottir, et al., 2019).

According to earlier studies, the outcomes for professionals related to flexible working (Ahonen et al., 2016), improved workflows (Collins et al., 2015; Simpson, 2013; Strudwick, Nagle, Kassam, et al., 2019) and higher levels of commitment and satisfaction with digital services (Ahonen et al., 2016; Collins et al., 2017; Gjellebæk et al., 2020; Kujala, Hörhammer, et al., 2019; Liebe et al., 2016; Mills et al., 2010). According to included studies, leadership in this context also increased professionals' informatics competency (Strudwick, Nagle, Morgan, et al., 2019) and development (Ennis‐Cole et al., 2018). Earlier studies showed that several different consequences related to patients; leadership seemed to advance patient‐centredness (Ahonen et al., 2016; Collins et al., 2015), improve the quality (Ahonen et al., 2016; Collins et al., 2015; Mills et al., 2010; Simon et al., 2013; Strudwick, Nagle, Morgan, et al., 2019), safety (Ahonen et al., 2016; Simon et al., 2013) and efficiency of care (Ennis‐Cole et al., 2018).

#### 4.2.4 Model case, borderline and contrary cases

We developed one model case to represent a real‐life example of leadership using the defining attributes discussed earlier. We also represented borderline and contrary cases (Table 4).

**TABLE 4:** Model, borderline and contrary cases of leadership in the context of digital health services

Model case	Borderline case *(closely resembles the model case but may lack at least some of the defining attributes)*	Contrary case *(clearly not an instance of leadership in the context of digital health services)*
Health care organization A has defined eHealth as its strategy and has thus implemented several different digital health services, such as EHRs, patient portals and online communication platforms. These services have been successfully designed and implemented in a workgroup that consisted of several different stakeholders, including top, clinical and IT management, staff, vendors and patients. One of the key persons in the implementation and development group is the chief nurse executive, with 25 years of experience, who is known for being *innovative and visionary* when it comes to digital health services. She/he is a *strong and effective leader* who has strategic eye *(strategic‐oriented)* what it comes to digital health services. Her/his *authoritativeness* is relevant to eHealth project's success.	Health care organization B has lately defined eHealth in its strategy and implemented some digital health services. The decision about digital health services is solely made by IT management and vendors, with just ratification from top managers. The organization has no chief medical or nursing informatics officers. Nurse executive B has attended to some education/training concerning digital health services, but rather concentrated on leading traditional clinical services than digital services. However, IT training has not been provided, for example, to educate staff to use new digital health services.	Organization C has not defined any strategy regarding eHealth, although it is aware that it has been part of the national programme for several years. However, they some must‐have IT solutions, such as EHRs, have been implemented, but the nurse executive C among other leaders acquiesced in the decision‐making with others. IT management selected the EHR to be implemented, which was ratified by top management. No employees or frontline leaders participated in EHR selection. Nurse executive C who was responsible for the implementation, did not take any training concerning the implementing solution. Implementation has been made rapidly and with insufficient resources. Training of the employees has happened at work. Nurse executive C is not very familiar with IT, politics related to it, or any risks that may be associated with it. She/he simply prefers to use e‐mail and leave everything else regarding technology to the IT department. IT decisions tend to fail to meet clinical practice needs. Nurse executive C has not discarded traditional models of bureaucratic leadership.
When implementing new digital health services, organization A has provided IT and software training for health care leaders in all leadership positions from top managers to frontline leaders, and nurse executive A has been attending to those (*competence oriented*). Due to the training, she/he has become skilled with informatics, and her/his skills are regularly evaluated by organization and herself/himself. She/he is familiar with fundamentals or IT, and she/he takes care that also other leaders at all levels are familiar with the fundamentals of IT, can analyse big data and use it for strategic decision‐making in an ethical way *(IT‐oriented)*. Leaders are trained to be critical towards the data that they receive from the decision‐support tools— – they evaluate the data *(knowledge‐oriented)*. Nurse executive A together with top managers has agreed that having informatics‐savvy staff is one strategic goal of organization A. Nurse executive A has encouraged and supported frontline leaders have arrange IT training for staff and regularly evaluate their IT skills *(supportive behaviour)*. She/he has also instructed frontline leaders to identify those professionals who either struggle with IT or are enthusiastic about it. For the first group they offer more support and training, while the professionals belonging to the latter group are identified as ‘champions’. Nurse executive A has adopted *transformational and change‐oriented behaviour*; she/he understands that implementation and usage of digital health services requires resources; enough resources are thus provided for IT projects *(resource allocator)*. Nurse executive A act as an example to her/his staff and remains visible, vocal; they are perceived as available to assist subordinates with IT when needed. In organization A, top managers are responsible for *decision‐making* concerning eHealth solutions and their implementation, whereas leaders on the frontline recruit clinical champions to advance the implementation of eHealth solutions. Nurse executive A understands that she/he needs to support all employees through frequent IT changes. She/he should recognize that her/his role as supporter is important when the organization is about to adopt or implement new digital solutions. She/he recognizes herself/himself as important information source *(informer)* and provides honest information concerning up‐coming digital services; aims to prevent negative rumours, instead justifying the benefits of digital health services from the perspective of the organization's strategy, and benefits for professionals and patients *(advocate)*. Nurse executive A asks frontline leaders observe how the digital service impacts on health care professionals' workflow and provide this information for middle and top managers. The digital services are developed so that they support professionals' workflow as well as possible and provide benefits for patients. Nurse executive A and other leaders engage staff and patients with the implementation and development process, and they work as *facilitators and collaborators* between different stakeholders. Nurse executive A utilizes social media for leadership and development purposes. When using social media, leaders are not bound to geographic locations or time zones, and they may even collaborate internationally *(being active)*.		

#### 4.2.5 Empirical referents

Empirical referents are measurable ways to demonstrate the occurrence of leadership in the study context. However, there exists no validated metrics to measure this complex phenomenon; instead, there is a metric that measures parts of it, namely, Nursing Informatics Competency Assessment for Nurse Leaders (Collins et al., 2017).

## 5 LIMITATIONS

This concept analysis has several limitations. Data extraction was completed by one researcher (although it was discussed in the research group). Few papers provided an implicit definition for leadership in the context of digital health services, and these definitions were context‐specific. The papers were conducted in affluent countries with high levels of digitalization; thus, the results might not be transferable to lower income countries. In addition, the database searchers had a limit of 10 years, which might have excluded some potential articles.

## 6 CONCLUSIONS

Our concept analysis creates a foundation for further research with its robust definition for leadership in digital health services. We encourage redefining and developing the term *e‐leadership* within the field. The constantly shifting context will doubtless influence leadership and leaders' behaviour. The increasing role of artificial intelligence may alter leadership in health care in the coming years—indeed, understanding leadership in the era of AI is surely a subject ripe for future study. To conclude, we urge use of the term e‐leadership in nursing research and in practice.

## 7 IMPLICATIONS FOR NURSING MANAGEMENT

The literature lacks a comprehensive understanding of leadership in the context of digital health services. Based on the results of our analysis, we created an initial framework of leadership, which may also be utilized when studying nursing leadership. As leadership, also nursing leadership develops over time and in different contexts. During the past decade, the rapid digitalization of health care has transformed nursing leadership.

Based on the results, there exist several different concepts through which leadership in our context may be defined. One of the most promising of these is the e‐leadership presented by Avolio et al. (2000); according to them, e‐leadership has been described as a socially influential process mediated by advanced IT to produce a change in individual/group/organizational thinking, feelings and performance. This definition fits with the attributes, antecedents and consequences represented in our concept analysis: for example, both our concept analysis and Avolio et al. (2014) definition include similar leadership behaviours; more specifically, we both highlight the importance of transformational leadership and effective e‐leaders. They also stated that effective e‐leaders understand the expectations of followers and communicate a clear vision, which was also seen as relevant within literature concerning health care leaders in the context of digital health services.

Second, the analysis reveals that leaders who are fully engaged in the context of digital health services seem to improve the implementation of those services; they release potential benefits or consequences to an organization. As stated earlier, digital health services implementation often fails (Herrmann et al., 2018; Öberg et al., 2018), and poor leadership might be one reason for that (Abbott et al., 2014; Mair et al., 2012). It might be that health care leaders, including nursing leaders, are ill‐prepared to handle digitalization; however, if they are fully engaged it seems—based on this work—that they might be more successful. This responsibility seems to fall especially on nurse leaders (Cowan, 2014), who based on our results work in an important role in a workgroup consisting of several different stakeholders (e.g., IT management, staff, IT vendors and patients).

Strudwick, Nagle, Kassam, et al. (2019) noted that leaders have to be more savvy with regard to IT. We recommend educating nurse leaders to become e‐leaders who adopt the necessary behaviours and roles and emphasize certain qualities when leading digital health services. Nurse leaders need to be visionary and use strategic thinking to develop existing and new digital solutions. Nurse leaders also need to listen to clinicians, evaluate their digital competence and provide support for frequent IT‐related changes. Nurse leaders need to advocate for clinicians and patients when developing digital health solutions. By becoming such e‐leaders, nurse leaders may increase the successful development and implementation of eHealth and benefit clinicians and patients.

## CONFLICT OF INTEREST

None.

## ETHICS STATEMENT

No ethical approval needed (no research with humans).

## Supporting information


**Appendix S1.** Search strategy

**Appendix S2.** Supporting Information

## Data Availability

Data sharing is not applicable to this article as no datasets were generated or analysed during the current study.
