# Supplementary material for: Leadership in the context of digital health services: A concept analysis
Source: J Nurs Manag. 2022 Aug 25;30(7):2763–80. doi: 10.1111/jonm.13763 (PMC10087820; doi:10.1111/jonm.13763)
Supplement: Supplementary file 1 — Appendix S1. Search strategy [file JONM-30-2763-s001.docx]

**Appendix A – Search strategy**

The following search strategies were applied on 31 November 2020:

**MEDLINE (via Ovid)**

| # | Searches | Results |
| --- | --- | --- |
| 1 | exp Telemedicine | 31,035 |
| 2 | exp Leadership | 41,770 |
| 3 | (eHealth or e-health) .ab,kf,kw,ti | 6,906 |
| 4 | (information technolog* or digital*).mp. and (health or medic* or nursing).ab,kf,kw,ti. | 366,268 |
| 5 | ”leader*”.ab.kf,kw,ti. | 77,905 |
| 6 | 1 or 3 or 4 | 68,262 |
| 7 | 2 or 5 | 98,458 |
| 8 | 6 and 7 | 1,316 |
| 9 | limit 8 to yr=”2010-Current” | 979 |

abstract, keywords f, keywords w, title

[mp=title, abstract, original title, name of substance word, subject heading word, floating sub-heading word, keyword heading word, organism supplementary concept word, protocol supplementary concept word, rare disease supplementary concept word, unique identifier, synonyms]

**Scopus**

| Search | Results |
| --- | --- |
| ( TITLE-ABS-KEY (telemedicine OR ehealth OR “e-health”) OR TITLE-ABS-KEY ( (“information technolog* OR digital* ) AND ( health OR medic* OR nursing ) ) AND TITLE-ABS-KEY (leader* ) ) AND PUBYEAR > 2009 | 1,767 |

**CINAHL**

| # | Searches | Results |
| --- | --- | --- |
| 1 | MH ”Telehealth+” | 25,740 |
| 2 | MH ”Telehealth+” OR (eHealth OR e-health) | 27,956 |
| 3 | (“information technolog* OR digital*) AND (health OR medic* OR nursing) | 28,968 |
| 4 | 2 OR 3 | 54,561 |
| 5 | MH “Leaders+” | 24,367 |
| 6 | MH “Leaders+” OR “leader*” | 103,145 |
| 7 | 4 AND 6 | 1,682 |
| 8 | Published date: 01012020-20211231 | 1,321 |
| 9 | Exclude MEDLINE records | 957 |

**ProQuest (ABI/INFORM)**

| # | Searches | Results |
| --- | --- | --- |
| 1 | noft(telemedicine OR ehealth OR “e-health”) AND noft(leader*)  Date: After December 31 2009 | 1,464 |
| 2 | noft(“information technolog* OR digital*) AND (health OR medic* OR nursing) AND (leader*)  Date: After December 31 2009 | 6,942 |
| 3 | 1 OR 2 | 8,208 |
| 4 | Limit to peer-review, scholarly journals | 335 |

noft: Anywhere expect full text.
